# Editorial: The fontan circulation: Problems and solutions

**DOI:** 10.3389/fped.2022.1087739

**Published:** 2022-12-01

**Authors:** R. Heying, Y. d’Udekem, M. Gewillig, J. Rychik

**Affiliations:** ^1^ Department of Pediatric Cardiology, University Hospitals Leuven, Leuven, Belgium; ^2^ The George Washington University School of Medicine and Health Sciences, Washington, DC, United States; ^3^ Division of Cardiovascular Surgery, Children’s National Hospital, Washington, DC, USA; ^4^ Division of Cardiology, The Children’s Hospital of Philadelphia, Philadelphia, United States

**Keywords:** univentricular heart, fontan circulation, cavopulmonary connection, follow-up, heart failure

**Editorial on the Research Topic** The Fontan Circulation: Problems and Solutions

The Fontan circulation has been developed as a strategy for multiple, unrepairable complex cardiac malformations in which there is only one effective ventricle. Almost 50 years later, the Fontan circulation is seen as an important achievement, resulting in good hemodynamic outcome for an otherwise untreatable heart disease. At the same time, complications occur due to challenges associated with a failing circulation due to the physiological features of chronically elevated systemic pressure and decreased cardiac output, obligatory features in the absence of a sub-pulmonary ventricle. As a consequence, patients are at risk for various complications including ventricular failure, organ failure, arrhythmias, protein losing enteropathy and plastic bronchitis. Despite recent improvements in tackling these complications, patients still face a high morbidity and mortality.

This special issue focuses on analyzing these challenges in a problem and potential solution framework. Fifteen manuscripts addressed various aspects of the Fontan circulation in children and adults. The contributions in this special issue articulate for practitioners and scientists what we can do today, and what we need to develop going forward.

## Problems and solutions: specifics of the Fontan pathophysiology and its failure

The Fontan operation is the palliative procedure of choice for single ventricle patients. It is achieved by connecting both caval veins directly to the pulmonary arteries without the interposition of a sub-pulmonary ventricle. This approach separates the systemic and pulmonary venous returns to the heart and markedly diminishes mixing of deoxygenated and oxygenated blood as well as reduces volume overload of the single ventricle. However, the resulting Fontan circulation, in which the systemic and pulmonary circulation are connected in series, is characterized by a distinct physiology with chronically elevated central venous pressure. Chronic preload deprivation of the single ventricle reduces cardiac output.

Currently, we are still facing a lack of comprehensive knowledge regarding Fontan circulatory physiology which translates into a lack of effective treatment options.

Van de Bruaene et al. (2022) shed light on underlying factors of Fontan failure and contributed with a comprehensive review drawing the focus on its most limiting factor: the pulmonary circulation. The systemic ventricle in a Fontan circulation lacks preload at rest and during exercise. Therefore, systemic venous return through the pulmonary circulation becomes the main determinant in the regulation of pulmonary blood flow and hence exercise capacity. Since there is no sub-pulmonary ventricle, even minimal changes in pulmonary vascular resistance cause significant changes in cardiac output. The authors suggest optimizing the Fontan conduit early in life to allow for sufficient pulmonary artery growth and later in life by stenting of the conduit to adult size. In particular, stenting of a hypoplastic left pulmonary artery if present is important. The value of physical activity-which introduces some degree of pulsatility to the pulmonary vasculature and improves filling of the ventricle with stiffness-reducing stretching- as well preventing the accumulation of risk factors for heart failure with a preserved ejection fraction phenotype (obesity, hypertension, diabetes) cannot be understated. In practice, pediatric cardiologists and congenital cardiac surgeons should always aim for the “perfect” Fontan circulation. The patient should maintain an active, healthy lifestyle avoiding weight gain and the adult congenital cardiologist should not accept suboptimal hemodynamics (i.e., AV valve regurgitation, undersized Fontan conduits, pulmonary artery hypoplasia).

Clinical presentation and hemodynamic phenotypes of Fontan failure are considerably variable. Kramer et al. (2022) developed an uncomplex yet remarkably accurate score to classify Fontan failure and late mortality in adult Fontan patients to allow a timely risk stratification. The score is based on hemodynamic, clinical and laboratory findings and is composed through analysis of a cohort of 198 adult Fontan patients with a median follow-up of about 20 years. Fifteen parameters were identified associated with Fontan failure and/or mortality. The accuracy to discriminate between patients with and without late Fontan failure as well as late mortality and survival was assessed in their cohort and patients with Fontan failure had a significantly higher median Fontan Failure Score compared to non-failing Fontan patients. Mortality associated with Fontan failure was substantial (48.1%). A prospective validation and likely refinement and calibration of the score in larger and preferably multi-institutional cohorts is still required.

## Problems and solutions: the respiratory system

Respiratory function plays a crucial role in Fontan circulation by having the systemic and pulmonary circulations in series. Ventilation strongly determines pulmonary blood flow and cardiac output at rest and with exercise. Laohachai et al. (2022) reviewed the impact of impaired lung function which is characterized by restrictive ventilatory patterns and a reduced lung volume. In addition, the authors report on the reduced skeletal and respiratory muscle strength in Fontan patients. Respiratory muscle training has shown potential promise to improve exercise capacity.

While respiration influences phasic pulsatility, it has a limited effect on the effective forward flow in the Fontan circulation. Interestingly, van der Woude et al. (2021) discussed the influence of respiration on blood flow based on insights gained from imaging-based clinical evaluations. In contrast to the healthy circulation, respiration is the main source of blood flow pulsatility in the Fontan circulation, whereas cardiac contraction mostly drives the effective forward flow rate. In particular hepatic venous flow, which contributes approximately 38% to the total Fontan tunnel flow, is strongly influenced by respiration. In essence, the higher blood flow during inspiration is countered by depressed flow rates during expiration. Since MRI examination is recommended every 2 years in Fontan patients, clinicians should be aware that most conventional MRI flow sequences do not capture the pulsatility of the blood flow as a result of respiration. Therefore, authors state that conventional ECG-gated PC-MRI acquisitions can be used for the measurement of clinical parameters based on net forward flow. Inclusion of respiratory phasic pulsatility in state-of-the-art patient-specific CFD models are recommended for evaluation of detailed, time-resolved hemodynamic metrics (e.g., wall shear stress and viscous energy loss rate), continuing to provide important insights for clinicians in the function of the Fontan circulation.

Pulmonary arteriovenous fistulae (PAVF) are one of the major complications after Fontan operation. Ohuchi et al. (2022) clarified the incidence, clinical characteristics and its influence on mortality. PAVF were present in 9.2% of 391 Fontan patients investigated by pulmonary artery angiography and/or contrast echocardiography during catheterization. Most patients (83%) showed a diffuse type of PAVF, associated with a significant decrease in mean arterial blood oxygen saturation compared to the non-PAVF group. Oxygen values in these patients decreased further corresponding to the postoperative stage from 90% at 1 year to 88% in the long-term follow-up of >25 years postoperatively. Authors highlighted that discrete-PAVF had no influence on SaO2 or mortality, whereas the presence of diffuse-PAVF caused hypoxia and had an adverse impact on all-cause mortality. The incidence of PAVF increases with patient age.

## Problems and solutions: lymphatic disorders

Patients with single ventricle palliation are susceptible to a variety of lymphatic abnormalities of both the thorax and abdomen, such as protein-losing enteropathy (PLE) and plastic bronchitis (PB). Imaging the central lymphatic system by dynamic contrast MR lymphangiography allows visualization of the lumbar lymphatic networks, the cisterna chyli and the thoracic duct. Utilizing these imaging tools, Dori et al. (2022) report on the management of patients with PLE and PB, potential life-threatening diseases affecting approximately 5%–15% of single ventricle patients. Conservative management of PLE involves use of diuretics including high-dose aldactone and sildenafil. The role of low-fat high-protein diet is less clear. Cardiac catheterization must be performed to determine hemodynamics and any reversible Fontan pathway obstruction. Fenestration creation or recreation can also be attempted. Patients that remain symptomatic can be started on enteric steroids, however this treatment strategy should be finite and limited due to the risk of serious side-effects and complications. Interventions for protein-losing enteropathy include embolization of hepatoduodenal and periduodenal lymphatic networks and procedures to lower pressure in the lymphatic system such as thoracic duct decompression.

Conservative management of PB involves inhaled bronchodilators, inhaled steroids and pulmonary vasodilators. If symptoms persist, medications aimed at breaking down the casts are used including inhaled tissue plasminogen activator. However, if active lymph leaking into the airway is present, lymphatic imaging and selective lymphatic duct embolization are needed. If symptoms in both entities, PLE and PB, persist despite lymphatic intervention, then thoracic duct decompression (interventional or surgical) or orthotopic heart transplant should be considered.

## Problems and solutions: the hepatic system

Hepatic dysfunction after Fontan operation can occur as a postoperative complication and might also develop long term. In a retrospective case control study of 409 patients after TCPC, Luo et al. (2022) identified an increased central venous pressure and intraoperative aortic cross-clamping as risk factors for postoperative and persistent liver dysfunction after day 7 postoperatively. Special attention to this patient group is suggested by the authors to prevent liver impairment.

Schleiger et al. (2021) reported on Fontan-associated liver disease in the adult population. They investigated the metabolic liver function with the liver maximum function capacity test (LiMAx®) in 39 patients and compared it to laboratory testing, elastography and ultrasound. The authors found preserved metabolic hepatic function in about 80% of the patients. Results of metabolic testing did not correlate to the severity of abnormal hepatic findings evaluated by sonography or laboratory analysis, indicating that while abnormalities are present, hepatic functionality remains relatively intact.

Both studies indicate that the development and progression of liver dysfunction in Fontan patients is not a linear or uniform process. Therefore, Schleiger and colleagues suggest that the diagnostic approach during follow-up should encompass a variety of modalities in order to obtain the most comprehensive picture. More work is necessary to determine the most valuable surveillance strategy in this domain.

## Problems and solutions: extra-cardiac systems

Organ dysfunction occurs beyond the heart, lungs, liver and gut in those with Fontan circulation. Ritmeester et al. (2022) present an interesting review on this subject and report on abnormalities in the nervous system, pituitary, kidneys, and musculoskeletal system. The thyroid axis may be affected by pituitary edema which is related to its unique vasculature. Therefore, awareness for potential hypothyroidism is important. Renal dysfunction is frequent and might be underestimated by creatinine based renal function testing due to myopenia as both lean muscle mass and bone mineral density are decreased in most of the Fontan patients. The assessment of cystatin C for renal function is recommended by the authors.

Little is known about the sexual health in patients after Fontan operation. Rubenis et al. (2021) assessed the sexual function of men with a Fontan circulation by performing a prospective, cross-sectional study based on the data from the Australian and New Zealand Fontan registry. Self-reported erectile function of 54 men with Fontan circulation was not significantly impaired when compared with historical controls, however sexual desire and overall satisfaction were reduced. The presence of erectile dysfunction was not correlated to the Fontan type or the New York Heart classification and the proportion of the cohort who had a prior pregnancy was congruent with population data.

## Problems and solutions: origins of neurodevelopmental challenges

Calderon et al. (2022) reviewed the findings that many Fontan patients present with impaired neurodevelopmental and mental health outcomes. Patients experience difficulties in areas of cognition related to attention and executive functioning, visual spatial reasoning and psychosocial development. A high risk for mental health morbidities, particularly anxiety disorders and depression, is present. Underlying alterations in brain processes may occur during fetal development which may be further influenced by hemodynamic parameters and perioperative factors. Variables such as multiple interventions requiring a prolonged hospital stay, is shown to be associated with adverse long-term neurodevelopmental outcome. The authors emphasize the benefit of early screening for neurodevelopmental deficits to initiate adequate support, thus allowing for early interventions and support to achieve the best potential outcomes.

## Problems and solutions: thromboembolic risk factors

The Fontan circulation is associated with an increased risk of thromboembolism. To prevent patients from pulmonary embolism or ischemic stroke Fontan patients are commonly treated with anti-platelet agents and/or anti-coagulants. Van Den Helm et al. (2022) addressed this topic and reviewed the nature of thromboembolism post Fontan surgery and the evidence for thromboprophylaxis management using anti-platelet and anti-coagulant agents. The authors highlight that the complex pathophysiology of the highly thrombogenic environment in a Fontan circulation is based on all 3 elements of Virchow's triad: endothelial cell dysfunction, abnormal blood flow and a hypercoagulable state caused by coagulation factor abnormalities. Further dysregulation of hemostasis can be caused by a tendency towards liver function abnormalities and associated serum protein imbalances. Subclinical cerebrovascular thromboembolic events are still underdiagnosed. Importantly, authors stated that the risk of thromboembolic events, mainly occurring in the venous system, is the highest in the first year post Fontan operation with a second peak 10 years post Fontan. Furthermore, the authors focus on the needs for an emerging consensus on prophylaxis. Fontan patients with no clinical complexities should receive life-long aspirin as thromboprophylaxis, whereas further anti-coagulation with vitamin K antagonists can be reserved for patients with special risk factors like previous thrombotic events or in the older Fontan patient. A comprehensive follow-up including monitoring and attention for bone mineral density should be provided. Currently, the use of new classes of oral anti-coagulants may hold promise but is not yet recommended as primary prevention due to the lack of evidence. Despite prophylaxis, a significant risk of thromboembolic disease post Fontan surgery remains.

## Problems and solutions: exercise tolerance

A markedly reduced exercise performance affects most Fontan patients. Reduced exercise capacity influences long-term quality of life and is associated with worse prognosis, although a subset of patients have high physical performance (“Super-Fontan”), which may represent a unique low-risk phenotype. Typical cardiopulmonary exercise testing response in Fontan patients includes a depressed peak heart rate (HR), elevated minute ventilation /carbon dioxide production slope (ventilatory inefficiency), reduced peak work rate and an increased breathing frequency.

Stating that patients with the best clinical outcome might provide important insights, Tran et al. (2021) studied 60 patients from the Australian and New Zealand registry with the “Super Fontan” phenotype defined as having a normal exercise tolerance. When compared to a Fontan group with impaired exercise capacity, the “Super-Fontan” phenotype is associated with a healthy weight, lower age at Fontan completion, better exercise self-efficacy and higher overall levels of sport and physical activity participation during physical development. This said, exercise capacity has significant implications on clinical outcome and survival. As the mechanisms underlying improvements in aerobic exercise capacity and the effects of exercise training on circulatory and end-organ function remain incompletely understood, Tran et al. (2022) focused on the need for developing adapted exercise programs. In this second manuscript, the authors present the planning of a large well designed, multi-center randomized controlled trial: The Fontan Fitness Intervention Trial which will investigate as a primary outcome the change in aerobic exercise capacity after a 4-month supervised aerobic and resistance exercise training program of moderate-to-vigorous intensity followed by an 8-month maintenance phase in children and adults. Results of this study are pending and have the potential to strongly impact clinical practice.

In line with this therapeutic strategy, Dirks et al. (2022) report on their innovative prospective study of home-based bicycle-ergometer and inspiratory muscle training for children and adults with Fontan circulation. After performing 90 min of endurance training per week in addition to inspiratory muscle training (30 breaths per day) for 10 months the authors observed significant increases in maximum relative workload and in maximum inspiratory and expiratory pressures. Peak VO2 values did increase significantly as compared to baseline in a subgroup analysis of teenage/adult patients while the subjective quality of life remained unchanged under a potential influence of COVID times. This study confirms that an individually adapted home-based training program is safe and is associated with improvements in exercise test variables.

## Future directions

The contributions of research efforts and reviews in this special issue on the Fontan circulation teach us to focus on principles of management and the identification of risk factors to improve patient outcomes. There is a great need for advancing multidisciplinary health care for this unique group. Special focus should be given to optimization of cardiovascular parameters pre-Fontan e.g., the pulmonary vasculature and the lymphatic vasculature. A comprehensive follow-up surveillance program post Fontan needs to pay attention to early discovery of organ dysfunctions. An important measure to prevent or reverse negative outcome and the development of complications appears to be the maintenance of a good exercise capacity with encouragement of physical activity and regular exercise training. According to Van de Bruaene et al. (2022) the areas in need of innovation to improve the Fontan circulation include:

- retraining of the pulmonary vasculature and ventricle
- using right-sided assist devices prior to transplant
- performing further research on implantable right-sided assist devices
- reducing the risk of AV valve surgery
-  determining novel pathways to improve the thrombo-inflammatory state in Fontan patients.

In this issue, perspectives are highlighted to improve long-term risk stratification and the implementation of new tools for future care to effectively improve the quality and duration of life of individuals with a Fontan circulation.

